# Supplementary material for: Handwashing and Detergent Treatment Greatly Reduce SARS-CoV-2 Viral Load on Halloween Candy Handled by COVID-19 Patients
Source: mSystems. 2020 Nov 17;5(6):e01074-20. doi: 10.1128/mSystems.01074-20 (PMC7743156; doi:10.1128/mSystems.01074-20)
Supplement: TEXT S1 [file mSystems.01074-20-s0001.docx]

**Supplemental Materials:**

Materials and Methods:

*Patient handling procedure:*

For each patient, four candy bags were prepared and labelled. Each individual biohazard bag contained two candies with the following identities: candy #1, a Haribo brand Halloween candy in a plastic package (from a Haribo Halloween candy mixed bag) and candy #2, either a mini Twix or a mini M&Ms or a mini Starburst or a mini Snickers.

Bags were divided into three patient handling conditions, and a fourth bag was gifted to each patient for their participation. The candy in each experimental bag was handled as follows: for the ‘unwashed’ bag, each candy was handled individually by patients with unwashed hands; for the ‘cough’ bag*,* each candy was handled individually by patients with unwashed hands that have been coughed into (patients were also are instructed to cough onto each candy to increase the likelihood of SARS-CoV-2 transfer); and for the ‘washed’ bag, each candy was handled individually by patients with washed hands (washed with soap for at least 20 seconds, as per CDC guidelines). Candy was transferred from the patients’ residences to the lab on ice in a cooler to be swabbed the same day.

*Post-handling treatments:*

In a cleaned, labelled 5000 mL plastic beaker, 20 mL of detergent (Method® Dish Soap, Clementine scent) containing SLS (a synonym for sodium dodecyl sulfate or SDS, commonly used in the lab) were mixed with 980 mL DI water. In a BioSafety Cabinet (BSC), one candy from each handling group from each individual participant was submerged in bulk in the prepared solution for no less than 1 minute with minimal agitation. Afterwards, candy was removed from the solution and the surfaces of the candy wrappers were swabbed. Candies that were untreated were swabbed right after being taken out of the transport bag.

*Candy wrapper swabbing:*

In a BSC, sterile flocculated swabs (Affordable IHC Solutions) soaked with 0.5% SDS were rubbed vigorously across the entire surface of the assorted candy wrappers, placed in their respective 1 mL matrix tube (ThermoFisher Scientific #3740TS), and broken at a designed breakpoint in order to be fully enclosed by the tube’s screwtop. The identity of each tube was tracked using the 2D barcode at the bottom of each tube and placed in a 96-well tube rack. Tubes were stored at -80 °C until ready for RNA extraction.

*Nucleic acid extraction:*

The 96-well rack containing tubes with swabs was thawed in room temperature water. When fully thawed, the 96-well tube rack was vortexed for 5 minutes to promote the suspension of viral particles from the swabs into the 0.5% SDS solution. Afterwards, 200 µL of the suspension buffer were transferred into a deep well extraction plate and processed using the Omega MagBind Viral DNA/RNA kit (Omega Bio-Tek #M6246) on the Kingfisher Flex (ThermoFisher Scientific) platform following manufacturers instructions, with an addition of 5 µL of MS2 phage to each well as an extraction control. The total time to perform the RNA extraction was approximately 60 minutes (15 minute setup, 45 minute automated extraction in Kingfisher).

*SARS-CoV-2 detection:*

*RT-qPCR (Multiplexed TaqPath)*

Viral gene detection assays were performed using the RT-qPCR-based TaqPath™ COVID-19 Combo Kit (ThermoFisher Scientific PN: A47814) on a QuantStudio 7 Pro with a 384-well sample block (ThermoFisher Scientific) according to the manufacturer’s protocol with the following modifications: 2 µL of purified RNA was added to a 1 µL reaction mix containing 0.75 µL TaqPath 4x Enzyme mix (ThermoFisher Scientific PN: A28523), 0.15 µL multiplex probe mix, and 0.1 µL nuclease free water, for a total reaction volume of 3 µL. The following cycling conditions were used: 25 °C for 2 minutes, 53 °C for 10 minutes, 95 °C for 2 minutes, 55 cycles of 95 °C for 3 seconds, and 60 °C for 30 seconds. The signal was measured at the end of each 30 second interval at 60 °C. Baseline determination and quantification cycle (Cq) signal determination were made using the Design and Analysis v2.4.3 software (Applied Biosystems) using the relative threshold (Crt) method. Positive calls for individual reporters were made following Table S1. The sample SARS-CoV-2 detection results were decided based on the criteria presented in Table S2.

*LAMP*

RT-LAMP was performed using WarmStart® Colorimetric LAMP 2X Master Mix with Antarctic Thermolabile Uracil-DNA Glycosylase (UDG) to reduce cross-contamination (NEB #M1804). The reaction was supplemented with 60 mM guanidine hydrochloride to run in multiplex. Previously published primer sets were used to target the envelope (E1) and nucleocapsid (N2) genes (*1-3*). Primer formulations were used at the NEB recommended concentrations. Aliquots of 5 µL sample RNA were added to each reaction. To control for false negatives, an additional reaction was run with a spike of 450 copies of synthetic SARS-CoV-2 RNA: ORF, E, N (ATCC® VR-3276T™). Reactions were incubated for 40 min at 65 °C, followed by 5 minutes at 4 °C. Change in color from pink (pH~8.8) to yellow or light orange (pH~5) was observed to identify positive samples.

*Viral load estimation*

SARS-CoV-2 viral load on candy was estimated through a linear regression model that relates expected genome equivalent (GE) copy numbers per µL of extracted nucleic acid (GE/µL) against measured Cq in RT-qPCR quantification from a limit of detection experiment that investigated detection of SARS-CoV-2 from surface swabs across different surface materials (wood, tile, plastic, metal) spiked with known concentrations of SARS-CoV-2 inactivated viral particles. The model grouped all surface materials together, used log-transformed expected GE copy numbers per µL(GE/µL), and had the following performance parameters: R=-0.9244, *p*=1.512e-33, and stderr=0.01778. (Table S3.)
