# Supplementary figures and images for: Handwashing and Detergent Treatment Greatly Reduce SARS-CoV-2 Viral Load on Halloween Candy Handled by COVID-19 Patients
Source: mSystems. 2020 Nov 17;5(6):e01074-20. doi: 10.1128/mSystems.01074-20 (PMC7743156; doi:10.1128/mSystems.01074-20)

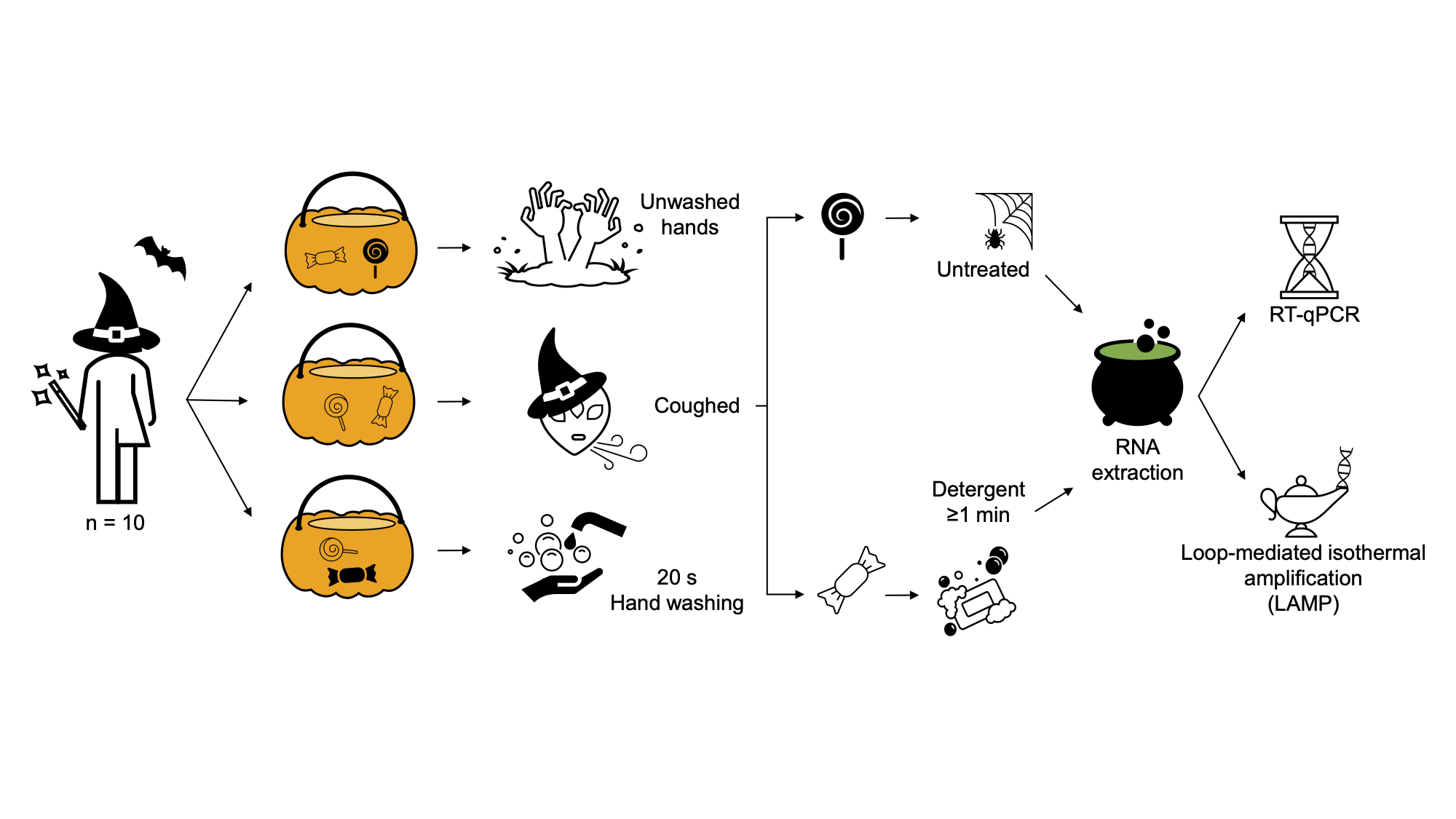

Supplement: FIG S1 [file mSystems.01074-20-sf001.tif]
