# Supplementary material for: Handwashing and Detergent Treatment Greatly Reduce SARS-CoV-2 Viral Load on Halloween Candy Handled by COVID-19 Patients
Source: mSystems. 2020 Nov 17;5(6):e01074-20. doi: 10.1128/mSystems.01074-20 (PMC7743156; doi:10.1128/mSystems.01074-20)
Supplement: TABLE S1 [file mSystems.01074-20-st001.docx]

| **Individual Target Passing Criteria** | | |
| --- | --- | --- |
| **Target** | **Cq Range** | **Cq Confidence** |
| ORF1ab | <40 | >0.7 |
| N Gene | <40 | >0.7 |
| S Gene | <40 | >0.7 |
| MS2 | <40 | >0.3 |
