# Supplementary material for: Handwashing and Detergent Treatment Greatly Reduce SARS-CoV-2 Viral Load on Halloween Candy Handled by COVID-19 Patients
Source: mSystems. 2020 Nov 17;5(6):e01074-20. doi: 10.1128/mSystems.01074-20 (PMC7743156; doi:10.1128/mSystems.01074-20)
Supplement: TABLE S2 [file mSystems.01074-20-st002.docx]

| **ORF1ab** | **N Gene** | **S Gene** | **MS2** | **Status** | **Result** |
| --- | --- | --- | --- | --- | --- |
| Neg | Neg | Neg | Neg | Invalid | NA |
| Neg | Neg | Neg | Pos | Negative | SARS-CoV-2 Not Detected |
| Only one target=Pos | | | Pos/Neg | Inconclusive | SARS-CoV-2 Inconclusive |
| Two or more Targets=Pos | | | Pos/Neg | Positive | SARS-CoV-2 Detected |
