# Supplementary material for: Handwashing and Detergent Treatment Greatly Reduce SARS-CoV-2 Viral Load on Halloween Candy Handled by COVID-19 Patients
Source: mSystems. 2020 Nov 17;5(6):e01074-20. doi: 10.1128/mSystems.01074-20 (PMC7743156; doi:10.1128/mSystems.01074-20)
Supplement: TABLE S3 [file mSystems.01074-20-st003.docx]

| **sample** | **subject** | **handling** | **Post-handling treatment** | **candy** | **Target** | **Cq** | **Estimated viral load (GE/µL)** |
| --- | --- | --- | --- | --- | --- | --- | --- |
| **SB_0149_cough_untreated** | SB_0149 | cough | untreated | SNICKERS | Orf 1ab | 31.25 | 3.82 |
| **SB_0149_cough_untreated** | SB_0149 | cough | untreated | SNICKERS | S Gene | 31.75 | 3.63 |
| **SB_0149_cough_untreated** | SB_0149 | cough | untreated | SNICKERS | N Gene | 31.47 | 3.93 |
| **SB_0149_unwashed_soap** | SB_0149 | unwashed | detergent | SNICKERS | N Gene | 39.28 | 0.29 |
| **SB_0149_unwashed_untreated** | SB_0149 | unwashed | untreated | TWIX | Orf 1ab | 33.14 | 1.88 |
| **SB_0149_unwashed_untreated** | SB_0149 | unwashed | untreated | TWIX | N Gene | 34.64 | 1.36 |
| **SB_0150_cough_untreated** | SB_0150 | cough | untreated | STARBURST | Orf 1ab | 33.74 | 1.5 |
| **SB_0150_cough_untreated** | SB_0150 | cough | untreated | STARBURST | S Gene | 35.38 | 1.02 |
| **SB_0150_cough_untreated** | SB_0150 | cough | untreated | STARBURST | N Gene | 34.92 | 1.24 |
| **SB_0150_unwashed_untreated** | SB_0150 | unwashed | untreated | M&M | S Gene | 34.26 | 1.51 |
| **SB_0150_unwashed_untreated** | SB_0150 | unwashed | untreated | M&M | N Gene | 35.57 | 1.0 |
| **SB_0152_cough_untreated** | SB_0152 | cough | untreated | M&M | Orf 1ab | 33.8 | 1.47 |
| **SB_0152_cough_untreated** | SB_0152 | cough | untreated | M&M | N Gene | 33.73 | 1.85 |
| **SB_0154_cough_soap** | SB_0154 | cough | detergent | SNICKERS | S Gene | 35.92 | 0.85 |
| **SB_0154_cough_soap** | SB_0154 | cough | detergent | SNICKERS | N Gene | 35.83 | 0.92 |
| **SB_0154_cough_untreated** | SB_0154 | cough | untreated | HARIBO | Orf 1ab | 29.77 | 6.66 |
| **SB_0154_cough_untreated** | SB_0154 | cough | untreated | HARIBO | S Gene | 30.59 | 5.44 |
| **SB_0154_cough_untreated** | SB_0154 | cough | untreated | HARIBO | N Gene | 30.07 | 6.26 |
| **SB_0154_unwashed_soap** | SB_0154 | unwashed | detergent | STARBURST | S Gene | 34.8 | 1.25 |
| **SB_0154_unwashed_soap** | SB_0154 | unwashed | detergent | STARBURST | N Gene | 35.68 | 0.96 |
| **SB_0154_unwashed_untreated** | SB_0154 | unwashed | untreated | HARIBO | Orf 1ab | 30.81 | 4.51 |
| **SB_0154_unwashed_untreated** | SB_0154 | unwashed | untreated | HARIBO | S Gene | 31.49 | 3.98 |
| **SB_0154_unwashed_untreated** | SB_0154 | unwashed | untreated | HARIBO | N Gene | 30.78 | 4.94 |
| **SB_0154_washed_soap** | SB_0154 | washed | detergent | M&M | S Gene | 33.89 | 1.72 |
| **SB_0154_washed_soap** | SB_0154 | washed | detergent | M&M | N Gene | 34.8 | 1.29 |
| **SB_0154_washed_untreated** | SB_0154 | washed | untreated | HARIBO | Orf 1ab | 32.27 | 2.61 |
| **SB_0154_washed_untreated** | SB_0154 | washed | untreated | HARIBO | S Gene | 33.82 | 1.76 |
| **SB_0154_washed_untreated** | SB_0154 | washed | untreated | HARIBO | N Gene | 32.59 | 2.7 |
| **SB_0155_cough_untreated** | SB_0155 | cough | untreated | HARIBO | N Gene | 35.6 | 0.99 |
| **SB_0156_cough_untreated** | SB_0156 | cough | untreated | HARIBO | S Gene | 35.23 | 1.07 |
| **SB_0156_cough_untreated** | SB_0156 | cough | untreated | HARIBO | N Gene | 33.66 | 1.89 |
| **SB_0156_unwashed_soap** | SB_0156 | unwashed | detergent | M&M | N Gene | 35.25 | 1.11 |
| **SB_0156_unwashed_untreated** | SB_0156 | unwashed | untreated | HARIBO | Orf 1ab | 32.84 | 2.1 |
| **SB_0156_unwashed_untreated** | SB_0156 | unwashed | untreated | HARIBO | S Gene | 33.93 | 1.69 |
| **SB_0156_unwashed_untreated** | SB_0156 | unwashed | untreated | HARIBO | N Gene | 33.5 | 2.0 |
| **SB_0157_cough_soap** | SB_0157 | cough | detergent | TWIX | S Gene | 35.85 | 0.87 |
| **SB_0158_unwashed_untreated** | SB_0158 | unwashed | untreated | HARIBO | Orf 1ab | 34.3 | 1.22 |
| **SB_0158_unwashed_untreated** | SB_0158 | unwashed | untreated | HARIBO | N Gene | 35.63 | 0.98 |
